# Localization of a Female-Specific Marker on the Chromosomes of the Brown Seaweed *Saccharina japonica* Using Fluorescence *In Situ* Hybridization

**DOI:** 10.1371/journal.pone.0048784

**Published:** 2012-11-07

**Authors:** Yu Liu, YanHui Bi, JunGang Gu, LiHua Li, ZhiGang Zhou

**Affiliations:** College of Aqua-life Sciences and Technology, Shanghai Ocean University, Shanghai, China; Leibniz Center for Tropical Marine Ecology, Germany

## Abstract

**Background:**

There is a heteromorphic alternative life in the brown seaweed, *Saccharina japonica* (Aresch.) C. E. Lane, C. Mayes et G. W. Saunders ( = *Laminaria japonica* Aresch.), with macroscopic monoecious sporophytes and microscopic diecious gametophytes. Female gametophytes are genetically different from males. It is very difficult to identify the parent of a sporophyte using only routine cytological techniques due to homomorphic chromosomes. A sex-specific marker is one of the best ways to make this determination.

**Methodology/Principal Findings:**

To obtain clear images, chromosome preparation was improved using maceration enzymes and fluorochrome 4′, 6-diamidino-2-phenylindole (DAPI). The chromosome number of both male and female haploid gametophytes was 31, and there were 62 chromosomes in diploid sporophytes. Although the female chromosomes ranged from 0.77 µm to 2.61 µm in size and were larger than the corresponding ones in the males (from 0.57 µm to 2.16 µm), there was not a very large X chromosome in the females. Based on the known female-related FRML-494 marker, co-electrophoresis and Southern blot profiles demonstrated that it was inheritable and specific to female gametophytes. Using modified fluorescence *in situ* hybridization (FISH), this marker could be localized on one unique chromosome of the female gametophytes as well as the sporophytes, whereas no hybridization signal was detected in the male gametophytes.

**Conclusions/Significance:**

Our data suggest that this marker was a female chromosome-specific DNA sequence. This is the first report of molecular marker localization on algal chromosomes. This research provides evidence for the benefit of using FISH for identifying molecular markers for sex identification, isolation of specific genes linked to this marker in the females, and sex determination of *S*. *japonica* gametophytes in the future.

## Introduction

*Saccharina japonica* (Aresch.) C. E. Lane, C. Mayes et G. W. Saunders ( = *Laminaria japonica* Aresch.) is a brown seaweed of high economic importance especially in East Asia, such as in China, Japan and South Korea, where it has been cultivated extensively for food and industrial alginate. China is by far the largest producer, and the production of *L*. *japonica* in China in 2009 rose sharply to 4.14×10^9^ kg wet weight [1], accounting for approximately 80% of the global production, over several decades. This has been attributed to both a fundamental understanding of its biology as well as the cultivation techniques and genetic breeding [2]–[5]. There are two heteromorphic alternative forms in *S*. *japonica*, macroscopic monoecious sporophytes (2n) and microscopic diecious gametophytes (n). The haploid female and male gametophytes differ from each other not only in morphology [6] and physiology [7], [8], but also in genetics. The female gametophytes can develop and produce eggs and even give rise to parthenogenetic sporophytes without fertilization [9]–[12] whereas the males produce sexual spermatozoids [13]. These discrepancies possibly result from the different genes or differential expression of genes between males and females. Accordingly, attempts have been made to identify sex-specific genes by construction and characterization of subtraction cDNA libraries using suppression subtractive hybridization [14], [15]. Unfortunately, no sex -specific genes were found in these studies.

At the same time, a sequence-characterized amplified region (SCAR) marker, FRML-494 (GenBank accession No. EU931619), related to the female gametophytes was developed [16] on the basis of a specific sequence (GenBank accession No. AB069714) of the *Marchantia polymorpha* L. Y chromosome. However, no cytological studies were conducted to further characterize the sex-specific marker. In order to utilize this putative sex-specific marker as a tool for sex identification, which otherwise was possible only through morphological identification at the early developmental stage of zoospore germination, additional studies are required to provide cytological evidence of sex linkage to this putative sex-specific marker. The objective of this study was to look into the specific relationship between the marker and female gametophytes and to determine chromosomal localization using Southern blotting and fluorescence *in situ* hybridization (FISH).

FISH was initially developed in the field of mammalian research [17] on the basis of the theory and protocol of *in situ* hybridization [18], [19]. Since the first reports of implementing the FISH technique in plant research, reported independently by Le et al. [20] and Schwarzacher et al. [21], this powerful tool has been widely used in genome analysis of higher plants (see the reviews [22]–[24]). FISH enables the physical mapping of DNA sequences on chromosomes during mitosis and meiosis. In spite of the widespread use of FISH in animals and higher plants, it has been rarely used in algae, possibly due to the difficulties in chromosomal preparation, large numbers of chromosomes and their relatively small sizes [25]. Well-spread chromosome preparation is thought to be an essential prerequisite for a successful application of FISH protocols [17], [26], [27].

The chromosome number of *S*. *japonica* has been examined by a number of researchers including Abe [28], Yabu [29], Tai and Fang [9], [30], Yabu and Yasui [31] and Zhou et al. [32], but there has not been a consistent conclusion because of the inconsistencies and difficulties in chromosome images. After comparing all the reports on kelp chromosomes, it is apparent that a sensitive staining method is needed to provide the best contrast and bright photographs because of the small sizes and nearly identical shapes of the kelp chromosomes [25], [30], [33], [34].

The interaction of 4′, 6-diamidino-2-phenylindole (DAPI) with DNA and polydeoxynucleotides has been extensively studied subsequently to this chemical being synthesized by Dann et al. [35]. It is now generally accepted that DAPI binds to DNA preferentially at AT-rich regions [36] in solution, forming highly fluorescent complexes. When excited with UV light at λ = 365 nm, the DNA-DAPI complex fluoresces a bright blue at 390 nm or >390 nm, while unbound DAPI and DAPI bound to non-DNA material may fluoresce a weak yellow [37]. DAPI, therefore, has been used as a cytochemical probe for nuclear DNA content measurement of algae [38]–[47]. However, DAPI was used in only a few reports for chromosomal observations [43], [48]. Therefore, the first step in this research was to prepare high quality kelp chromosomes stained with DAPI to better visualize them. Subsequently, the developed putative sex-specific marker FRML-494 (GenBank accession No. EU931619) from the female and male gametophytes (n = 10 each) of *S*. *japonica* [16] was mapped to the kelp chromosomes using the FISH technique. Here we present our results and experience with mapping of the putative sex-specific marker to chromosomes.

## Materials and Methods

### Alga and Cultures

The Rongfu strain of *S*. *japonica* was selected as the plant material in the present research, and its gametophyte clones germinated from zoospores were isolated according to cell sizes under a microscope and cultured under vegetative growth conditions of 30 µmol photons/m^2^ ·s at 17±1°C with a photoperiod of 12∶12 light∶dark (L∶D), as described previously [49]. PES medium [50] was replaced once every two weeks.

The diploid sporophytes were cultivated with the sporelings raised using the Rongfu strain gametophyte clones, as described by Li et al. [51]. The female and male gametophytes were mixed and cut into several-celled fragments with a blender and were washed thoroughly with distilled seawater. The gametophyte fragments suspended in seawater were poured into 500 mL glass beakers and kept motionless to allow the gametophytes to settle down on a palm rope substratum. When the sporelings grew to 0.8–1 cm in length, the palm ropes were taken out to the open sea. After cultivation at sea for about two months, the young sporophytes (about 1 m in length) were sampled for this study.

### DNA Extraction

One gram of samples of sporophyte tissue was digested in an enzymatic solution (8 mL) containing seawater, 0.8 M mannitol, 50 mM trisodium citrate, 1% (w/v) cellulase (Sigma-Aldrich, St. Louis, MO, USA), 0.5% (w/v) macerozyme R-10 (Yakult, Tokyo, Japan), and 0.2 U/mL of the prepared abalone alginate lyase for 8 h at 14°C to eliminate polysaccharide contaminants, as described previously [52]. After digestion, unicells were filtered from the undigested debris with a 240-mesh (60 µm) nylon net and centrifuged at 3 000×g for 5 min, and then the unicell pellets were washed five times with sterilized seawater in order to remove possible contamination from other organisms.

Genomic DNA was extracted separately from the freshly harvested gametophytes and the prepared unicells from the individuals of sporophytes according to the modified cetyltrimethyl ammonium bromide (CTAB) method as described by Hu and Zhou [52].

### Identification of the FRML-494 Marker in Gametophytes and Sporophytes

Polymerase chain reaction (PCR; 20 µL) solution contained 50 ng of genomic DNA as a template isolated from the female and male gametophytes and sporophytes of *S*. *japonica*, 1×reaction buffer, 2.5 mM of MgCl_2_, 100 µM of dNTPs, 0.2 µM of each primer and 1.0 unit of *Taq* DNA polymerase (TaKaRa, Dalian, China). Amplification of the genomic DNA with a pair of the reported primers P51 (forward 5′-AAGACAAGCGGGTGAACTCAGCGAGGTCT-3′, and reverse 5′-ACACTGGACATCGCATCGTCGATCAGTGT-3′) [16] was programmed using 1 cycle containing pre-denaturation at 94°C for 5 min, and 30 cycles with denaturation at 94°C for 30 s, annealing at 62°C for 45 s, and extension at 72°C for 1 min in a Mastercycler Gradient (Eppendorf, Hamburg, Germany). A final extension was performed at 72°C for 10 min. The amplified product was resolved on a 1.2% low-melting-point agarose gel for DNA recovery, cloning and sequencing. The target product was purified using a UNIQ-10 column DNA gel extraction kit (Sangon, Shanghai, China) and ligated into a pMDT-19 vector (TaKaRa, Dalian, China), and the latter was subsequently transformed into *Escherichia coli* JM109 competent cells (Leihao, Shanghai, China). Sequencing was performed in Sangon (Shanghai, China) using the dideoxynucleotide chain-termination method with an Applied Biosystems 3730 sequencer (Applied Biosystems, Foster City, CA, USA).

### Southern Blot

Aliquots of isolated DNA (approximately 20 µg per sample) were digested to completion at 37°C for 4–6 h independently with *Not*I and *Xba*I, which could not digest the FRML-494 marker. The digested DNA samples were fractionated on a 1.0% agarose gel, blotted onto a positively charged nylon membrane (Pall, Exton, PA, USA) and hybridized with the FRML-494 marker as a probe. The probe was labeled with biotin-dUTP using a North2South Biotin Random Prime Labeling Kit (Thermo, Rockford, IL, USA). Southern blots were performed following the manufacturer's instructions. Hybridized bands were detected using a North2South Chemiluminescent Hybridization and Detection Kit (Thermo, Rockford, IL, USA) and signals were visualized by exposure to XBT-1 film (Kodak, Rochester, USA) at room temperature for 90 s.

### Chromosome Preparation

The fresh gametophytes and sporophytic tissues of this kelp were separately treated with 0.02% colchicine for 8–10 h at room temperature and were washed three times in distilled seawater. The samples were then fixed in a freshly prepared Cannoy's fixative solution (100% ethanol: acetic acid, 3∶1, v/v) for 24 h [53] followed by a wash in distilled water three times. The fixed gametophytes or sporophytic tissues were digested with a gentle stir in a multi-enzyme solution I (cellulase: pectinase (Sigma-Aldrich, St. Louis, MO, USA): macerozyme R-10, 2∶1∶1, v/v) for 18 h at 37°C, and were harvested with a centrifuge at 13 000×g for 1 min. The supernatant was discarded and the pellets were re-digested in a multi-enzyme solution II (cellulase: macerozyme R-10: abalone alginate lyase, 2∶1∶2, v/v) at 37°C overnight, where the crude alginate lyase was extracted from abalone hepatopancreas as described by Hu and Zhou [52]. The samples were washed three times in 70% ethanol to remove the enzyme solution and were centrifuged at 13 000×g for 3 min to discard the ethanol. The pellets were re-suspended in acetic acid, and 6.5 µL cell suspensions were dropped on slides. The slides were screened under a phase contrast microscope for chromosomes at mitotic metaphase. The well-prepared slides were submitted to FISH detection and DAPI counterstaining.

### DAPI Counterstaining and FISH

For the FISH technique, an unlabeled 494 bp-long SCAR marker FRML-494 probe was generated by PCR with the female gametophyte genomic DNA as a template. PCR was carried out using the pair of primers P51 as mentioned above. The probe was labeled with Texas Red-5-dUTP (PerkinElmer, Boston, MA, USA) by nick translation using an ADVANCE™ Nick Translation Kit (Sigma-Aldrich, St. Louis, MO, USA) under the guidance of the manufacturer's instructions.

The FISH procedure followed Schwarzacher [26] with slight modifications. The chromosomes on slides were ultraviolet (UV) light cross-linked twice in a UV cross-linker BLX-E254 (Vilbert Lourmat, France) at 254 nm for 15 s each time, followed by the addition of 8 µL of hybridization buffer (0.9 M NaCl, 20 mM Tris-HCl, pH 7.2, 0.01% SDS) containing 5 ng/µL of labeled FRML-494 probe. The slides were heated to 100°C in a water bath for 5 min and then transferred to a 55°C air oven for 12 h for hybridization to occur. Post-hybridization washes of the slides were carried out in a slightly more stringent solution of 2×SSC (0.3 M NaCl, 0.03 M sodium citrate, pH 7) at 42°C for 10 min to remove unbound, nonspecifically bound, or weakly hybridized probes.

The chromosomes were counterstained with 15 µL DAPI in a VectaShield mounting medium (Vector Laboratories, Burlingame, CA, USA) for 5 min incubation. Slides were stored in the dark before microscopic analysis.

### Microscope Imaging and Data Analysis

Chromosome preparations were studied and photographed under an Olympus BX61 epifluorescence microscope (Tokyo, Japan) using a 360 nm filter. Hybridization signals from Texas Red were subsequently recorded on the single photograph using a 470 nm filter by the second exposure. The images were captured with an Olympus DP-71 digital camera using the DP controller program and the chromosomes were measured with Image-Pro Plus software. Chromosome sizes and localization of the FRML-494 marker were measured digitally in at least five metaphase plates, and the software Microsoft Excel was used for calculations.

The karyotypic patterns of the gametophytes were edited using Adobe Photoshop software (ver. 3.0) based on the size of chromosomes, and the color contrast and brightness uniformity were treated using this software.

## Results

### Specificity and Heredity of the FRML-494 Marker

**Figure 1:**
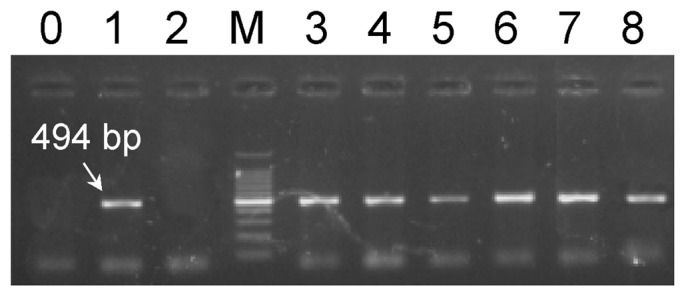
Electrophoresis profile of amplified products of the FRML-494 marker. PCR amplification using the pair of primers P51 in the female (lane 1) and male (lane 2) gametophytes and the crossbred sporophytes (lanes 3–8) of *Saccharina japonica* (strain Rongfu) showing the FRML-494 marker (as indicated by the arrow) was only present in the female gametophytes and sporophytes. Lane M, 100 bp DNA ladder marker (Generay, Shanghai, China). Lane 0, control with H_2_O instead of DNA.

A sex marker, FRML-494 (GenBank accession No. EU931619), was previously reported to be present only in the female gametophytes of *S*. *japonica* [16]. In this study, FRML-494 was detected to be present in both female gametophytes and randomly selected sporophyte individuals by crossbreeding of the female and male gametophytes, but was absent in the male gametophytes (Figure 1). Sequencing analysis showed that these existing bands were completely identical in both sequence and size, as previously reported [16]. This indicated that the FRML-494 marker related to the females was inheritable without any changes at the locus, thus being preserved as a whole in the crossbred sporophytes.

**Figure 2:**
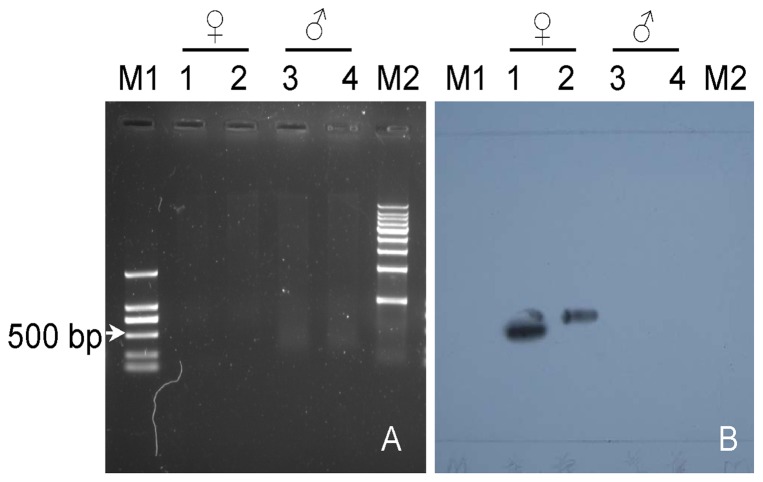
Southern blot of the labeled FRML-494 marker. Ethidium-bromide-stained agarose gel (A) of *Not*I- (lanes 1 and 3) and *Xba*I- (lanes 2 and 4) digested genomic DNA from the female (lanes 1 and 2) and male (lanes 3 and 4) gametophytes of *S*. *japonica*. Southern blot (B) of *Not*I- (lanes 1 and 3) and *Xba*I- (lanes 2 and 4) digested genomic DNA hybridized with the biotin-labeled FRML-494 marker. Lanes M1 and M2 show the D2000 and 1 kb DNA ladder molecular standard, respectively, (Tiangen, Beijing, China).

Southern blot profiling (Figure 2) showed that the labeled FRML-494 probe was able to hybridize only to DNA from the female gametophytes, but not to DNA isolated from the male gametophytes, confirming that the FRML-494 marker was specific to the female gametophytes.

### Determination of Chromosome Number and Chromosomal Characteristics

**Figure 3:**
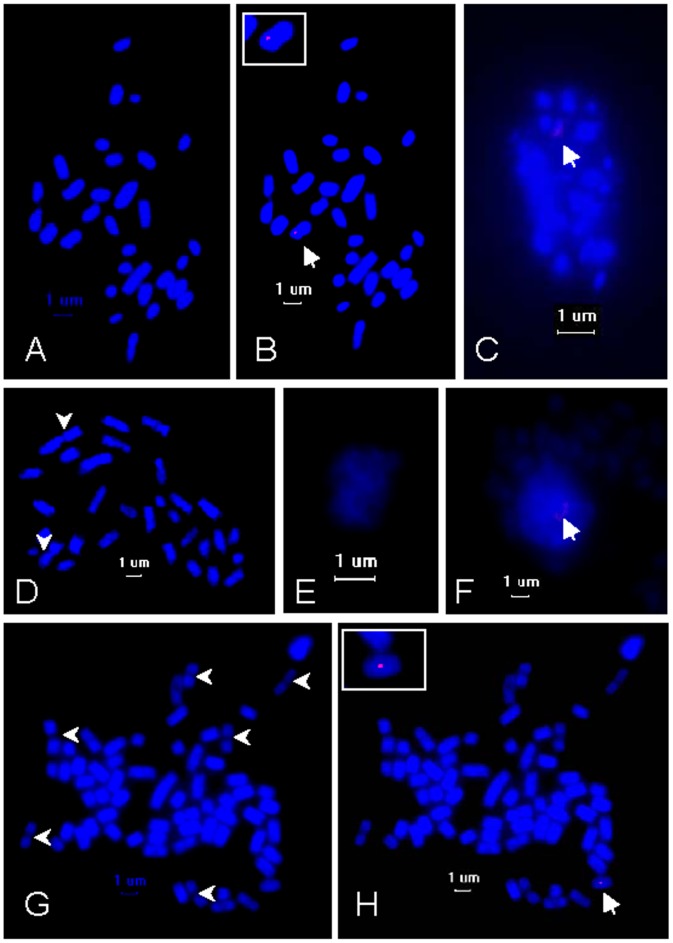
FISH of the FRML-494 marker (red) on *Saccharina japonica* chromosomes counterstained with DAPI (blue). (A) Metaphase chromosomes of the female gametophytes (n = 31); (B) hybridization signal of the FRML-494 marker (red) as indicated by the arrow on the same spread as image A; the inset is the enlarged chromosome where this marker was located; (C) an interphase nucleus of the female gametophytes with hybridization signal (red) as indicated by the arrow; (D) metaphase chromosomes of the male gametophytes (n = 31); (E) an interphase nucleus of the male gametophytes without a hybridization signal; (F) an interphase nucleus of the sporophytes with a hybridization signal (red) as indicated by the arrow; (G) metaphase chromosomes of the sporophytes (2n = 62); (H) hybridization signal (red) as indicated by the arrow present on only one metaphase chromosome of the same spread as image G; the inset is the enlarged chromosome where this marker was located; and the arrowheads in images D and G show the constriction of the chromosomes.

In order to prepare high quality chromosome slides, the sporophyte tissues or filamentous gametophytes of *S*. *japonica* were pretreated with colchicine for 8–10 h and macerated with multi-enzyme solutions for one to two days. Subsequently, the well-spread slides were obtained by dropping the pretreated materials in acetic acid over the slides at approximately 30 cm in height. Clear digital images (Figure 3A, D and G) of the smeared kelp chromosomes stained with DAPI were then captured using fluorescent microscopy. Counting chromosomes after DAPI staining was more feasible since the well-spread chromosomes stained using the DAPI solution gave a stronger contrast and brighter signals (Figure 3A, D and G). In some images (e.g. Figure 3D and G), the sharp constriction where a centromere of a metaphase chromosome was located was also visible. The chromosome numbers of the haploid gametophytes were 31 no matter whether they were from females (chromosome number ranged from 25 to 31, 15 out of 30 images with 31 chromosomes) (Figure 3A) or males (chromosome number ranged from 28 to 31, 12 out of 20 images with 31 chromosomes) (Figure 3D), and those of the diploid sporophytes were 62 (chromosome number ranged from 52 to 62, 8 out of 12 images with 62 chromosomes), as illustrated in Figure 3G. It was noted that not all the observed nuclear number was 31 or 62, because there were chromosome variances at different cytological phases observed.

The average absolute length (Table 1) of the chromosomes assayed in 5 images of the female slides was from 0.77 µm to 2.61 µm with 5 chromosomes longer than 2 µm and 3 shorter than 1 µm, whereas that from the males ranged from 0.57 µm to 2.16 µm with only one longer than 2 µm and 7 shorter than 1 µm. It seemed that the size of the female chromosomes was slightly larger than that of the corresponding males but there was no significant difference between their relative lengths, both of which varied approximately from 1.5% to 5.5% of the total chromosome length (Table 1). These data excluded the possibility that the female nucleus had a very large X chromosome. Apart from 3 to 6 spherical chromosomes in female gametophytes, the others were rod-like in shape (Figure 3A, D and G); such a karyogram of the gametophytes can be ranked according to their sizes (e.g. Figure S1A′, B′ and C′). Because there was not enough banding (e.g. Giemsa C- and G-banding, silver banding) information, the chromosome complement based on the image of sporophyte chromosomes (Figure 3G) could not be worked out.

**Table 1:** Length of chromosomes prepared from the male or female gametophytes of *Saccharina japonica*.

Chromosome No. only according to the decreasing size	Length of chromosomes from the male gametophytes (means±SD, n = 5)		Length of chromosomes from the female gametophytes (means±SD, n = 5)	
	Absolute (µm)	Relative (% of total ones)	Absolute (µm)	Relative (% of total ones)
I	2.160±0.010	5.53	2.612±0.113	5.57
II	1.983±0.149	5.08	2.397±0.115	5.11
III	1.902±0.128	4.87	2.149±0.150	4.58
IV	1.792±0.140	4.59	2.109±0.159	4.50
V	1.724±0.162	4.42	2.014±0.178	4.29
VI	1.636±0.138	4.19	1.877±0.220	4.00
VII	1.607±0.130	4.12	1.784±0.121	3.80
VIII	1.481±0.140	3.79	1.751±0.151	3.73
IX	1.432±0.134	3.67	1.724±0.162	3.68
X	1.387±0.131	3.55	1.686±0.169	3.59
XI	1.324±0.0698	3.39	1.657±0.186	3.53
XII	1.316±0.0712	3.37	1.619±0.163	3.45
XIII	1.299±0.0580	3.33	1.588±0.194	3.39
XIV	1.267±0.0604	3.25	1.567±0.193	3.33
XV	1.222±0.0719	3.13	1.513±0.192	3.23
XVI	1.184±0.0489	3.03	1.463±0.220	3.12
XVII	1.174±0.0499	3.01	1.441±0.215	3.07
XVIII	1.146±0.0679	2.94	1.419±0.216	3.03
XIX	1.115±0.0587	2.86	1.301±0.213	2.77
XX	1.108±0.0584	2.84	1.289±0.201	2.75
XXI	1.089±0.0575	2.79	1.257±0.186	2.68
XXII	1.072±0.0432	2.75	1.237±0.185	2.64
XXIII	1.039±0.0295	2.66	1.217±0.176	2.59
XXIV	1.028±0.0285	2.63	1.202±0.183	2.56
XXV	0.951±0.0483	2.44	1.158±0.187	2.47
XXVI	0.927±0.0578	2.37	1.120±0.197	2.39
XXVII	0.867±0.0818	2.22	1.098±0.207	2.34
XXVIII	0.826±0.0484	2.12	1.041±0.169	2.22
XXIX	0.776±0.0606	1.99	0.979±0.181	2.09
XXX	0.638±0.0243	1.63	0.853±0.184	1.82
XXXI	0.567±0.0153	1.45	0.773±0.142	1.64

### FISH of the FRML-494 Marker on One Unique Chromosome of the Kelp

To study the localization of the FRML-494 marker, 30 nuclei of the female samples were identified at metaphase and examined with FISH. Of these nuclei, 16 nuclei could be detected with a fluorescent signal present on one chromosome (Figure 3B). If the female interphase nuclei were examined, 25 of 30 nuclei showed a distinguishable fluorescent signal as shown in Figure 3C. On the contrary, there was no hybridization signal detected in all 20 of the examined male nuclei at metaphase (Figure 3D) or interphase (Figure 3E). These results strongly suggested that the FRML-494 marker was specifically present and localized on one chromosome of *S*. *japonica* female gametophytes without any homologous locus on the male chromosomes.

To confirm whether the chromosome where the FRML-494 marker was located was the same, three different images (Figure S1) were used as examples for the chromosome rearrangement and were edited using Adobe Photoshop software. Illustration showed that the FRML-494 marker only appeared on the 10^th^ chromosome (with a relative length from 3.56% to 3.67% in these 3 images), which was shown according to the decreasing size of the chromosomes from the female gametophytes. The shape of the chromosome where the FRML-494 marker was located and the site of the hybridization signal on this chromosome were similar as well. In addition, the FISH image (Figure 3F and H) from the diploid sporophyte nuclei showing the presence of a hybridization signal suggested that this marker was unique, too. So this FRML-494 marker was a female chromosome-specific cytogenetic DNA marker, as suggested by Jiang and Gill [23].

## Discussion

In this study, for the first time, we determined the inheritance and chromosomal location of a previously reported [16] putative sex-specific marker, FRML-494, in *S*. *japonica* by FISH. In addition, we provided indisputable evidence for chromosomal number and morphological characteristics of the kelp chromosomes through improved techniques including multi-enzyme treatment and DAPI staining. The use of FISH in plants lags considerably behind its applications in cytogenetics of human and other animal systems. A major factor, as suggested by Griffor et al. [54], contributing to the difficulty in plants was obtaining mitotic and meiotic chromosomes free of cell wall material. In general, a high quality chromosome preparation should be well spread and flat, and should have plenty of chromosomes with good morphology. Therefore, high quality material from *S*. *japonica* is an essential prerequisite, as suggested by Zhang and Friebe [27], for FISH of the FRML-494 marker on kelp chromosomes.

### Characteristics of *S*. *japonica* Chromosomes as Revealed by DAPI Staining

In most of the publications [29]-[31], [33], [34], [55] about chromosome preparations in the genus *Laminaria* or *Saccharina*, the samples were usually squashed without any treatment for chromosome preparation after fixation in Carnoy's solution. If the kelp gametophytes were digested with a multi-enzyme solution containing cellulase and pectinase, the preparation of chromosomes was reported to be improved for visualization [32]. Regarding the substantial existence of alginate in cell walls or middle lamella between two neighboring cells of the kelp, the crude alginase extracted from the abalone hepatopancreas was added to the multi-enzyme solution in the present research. Meanwhile, fluorochrome DAPI was used instead of the routine dyes, such as haematoxylin, orcein and carmine, for chromosome specific staining in cytological research of algae. In comparison with the published chromosome micrographs of kelp [29]–[32], the present chromosome images (Figure 3) displayed more contrast and were brighter without any noisy background, suggesting that such a chromosome preparation and staining was a successful protocol for karyotyping research of kelp. Furthermore, this result also showed that DAPI was a sensitive and effective dye for chromosome visualization in algae, such as *S*. *japonica* with small sizes and large numbers, although Lewis [25] thought the choice of stain was not critical since all the often-used dyes resulted in specific staining of chromosomes. To date, this is one of the few successful applications of DAPI in algal staining for the observations of chromosome shape and number [43], [48].

The chromosome images clearly illustrate that the chromosome numbers are 31 and 62, respectively, for the haploid (Figure 3A and D) and diploid (Figure 3G) plants of this kelp, which is in agreement with a report by Zhou et al. [32] and close to the result (haploid chromosome number was 32) by Yabu and Yasui [31], although the chromosome numbers have earlier been reported to be 22 and 44 [29], [30] for gametophytes and sporophytes, respectively. If the basic numbers of chromosomes are 8 or more in Laminariales [25], most taxa of brown algae hypothetically evolved the accompaniment of some aneuploidy from these basic numbers. In this case, the present research suggests that *S*. *japonica*, *S*. *latissima* (or *Laminaria saccharina*), *L*. *digitata*, *L*. *hyperborean* (Gunn.) Fosl. and *L*. *ochroleuca* Bachelot de la Pylaie are possibly at a similar evolutionary stage because they possess the same chromosome numbers in the haploid gametophytes [33], [34], although these kelps are now regarded as belonging to two different genera [2].

Most chromosomes of this kelp range from 1 µm to 2 µm in length (Table 1), so they are regarded as small chromosomes with a rigid field [56]. The ratios of the longest chromosome to the shortest are 3.81∶1 and 3.38∶1 in males and in females, respectively, showing that the kelp chromosomes belong to Type B chromosomes according to the criteria set by Stebbins (the ratio ranges from 2∶1 to 4∶1) [57]. In respect to the possible condensation of chromosomes during the preparation, the supplied relative lengths of female and male chromosomes (Table 1) demonstrate that there is no apparent distinction between males and females. Such a very large X chromosome found by Evans [33], [34] and Yasui [55] was not found to be present in the females (Figure 3A and Table 1), which is consistent with the conclusions drawn by Yabu [29], Tai and Fang [30], Yabu and Yasui [31] and Zhou et al. [32]. It seems that there is no sex chromosome in the kelp. On the contrary, the 1∶1 ratio of female to male gametophytes developed from the released meiospores [58] and all the female offspring from the parthenogenetic sporophytes [9]–[12] seemingly predict that the kelp has a sex chromosome. Therefore, the sex chromosome is supposed to be homomorphic with autosomes in both size and shape, as suggested by Fang et al. [10].

### Specific Localization of the FRML-494 Marker on Kelp Chromosomes

After chromosome preparation of high quality from *S*. *japonica*, the putative sex-specific marker, FRML-494 [16], was successfully localized within the kelp chromosomes (Figures 3 and S1). Taking the Southern blot (Figure 2) and FISH images (Figure 3B and H) together, it was concluded that this marker was female-specific and its locus was on one unique chromosome of the female gametophytes, both of which implies that this marker is a female chromosome-specific DNA sequence, as suggested by Jiang and Gill [23]. The median relative size of this chromosome on which the FRML-494 marker is located (Figures 3B and S1) indicates that it is not the very large X chromosome, as observed by Evans [33], [34] and Yasui [55]. Based on the absence of the very large X chromosome in the females, as discussed above, and the specific relationship between the FRML-494 marker and the female chromosomes, it is supposed that sex determination genes possibly exist on different chromosomes; i.e., multiple sex chromosomes, rather than a large chromosome as suggested by Fang et al. [10]. Therefore, whether the chromosome on which this maker is localized is a sex chromosome needs to be answered in the future.

Using FISH of the FRML-494 marker, it was noted that not all the metaphase female nuclei showed hybridization signals, and the hybridization signal in the FISH images (Figure 3B and H) was not bright enough either. This is because the probe was only 494 bp in length, which binds less fluorescent molecules rather than longer probes. Although probes <1 kb in length and even as small as 250 bp could be visualized through a fluorescence microscope in plant [59], [60] and human [61] chromosome preparations, respectively, the region of interest on the chromosome wrapped inside the superhelical structure allowed less access, especially for the short probes, thus possibly reducing the reproducibility of FISH [23], [62]. Since the chromosomes at interphase are decondensed and therefore stretched such that the target regions are easily exposed to the probes for FISH [63], the interphase nuclei (Figure 3C, E and F) can be regarded as references. Most of the female interphase nuclei (83.3%) showed the hybridization signals (Figure 3C) but no hybridization signal was present in either 20 metaphase (Figure 3D) or 20 interphase (Figure 3E) nuclei of the male gametophytes. Taking these data and Southern blot profiles (Figure 2) together, the FISH images (Figures 3 and S1) are convincing. If a co-existing sequence, such as the 45S rRNA gene (see the review [24]), is used as a probe to hybridize both males and females, it would be more helpful to support our results, and this approach will be attempted in the future.

In brief, after multi-enzyme maceration of cell walls, the kelp chromosome preparation can meet the needs of the FISH technique. The FRML-494 marker is successfully localized on one unique female gametophyte chromosome counterstained with DAPI. If the FRML-494 marker is genetically linked to the kelp female gametophytes, using this established FISH technique can surely help not only to distinguish the females from the males of this kelp, but also to clarify whether a kelp sporophyte is produced by crossbreeding or by monogenetic reproduction, although there is no distinct variation between the female and male karyograms, as discussed above. In addition, with the help of FISH, the physical mapping can be constructed so that the genome analysis, molecular breeding research and even the cytogenetical investigation of sex differentiation in *S*. *japonica* can be fast and promoted further.

## Supporting Information

Figure S1 **FISH images and their corresponding karyograms.** FISH of the labeled FRML-494 marker (red) on metaphase chromosomes of the female gametophytes counterstained with DAPI (blue). A′, B′ and C′ are the ordered chromosomes of FISH images of A, B and C, respectively, prepared with Adobe Photoshop by decreasing size in length. Arrows indicating the localization of the FRML-494 marker on one chromosome of the female gametophytes.
(TIF)
