# Supplementary figures and images for: Localization of a Female-Specific Marker on the Chromosomes of the Brown Seaweed Saccharina japonica Using Fluorescence In Situ Hybridization
Source: PLoS One. 2012 Nov 7;7(11):e48784. doi: 10.1371/journal.pone.0048784 (PMC3497718; doi:10.1371/journal.pone.0048784)

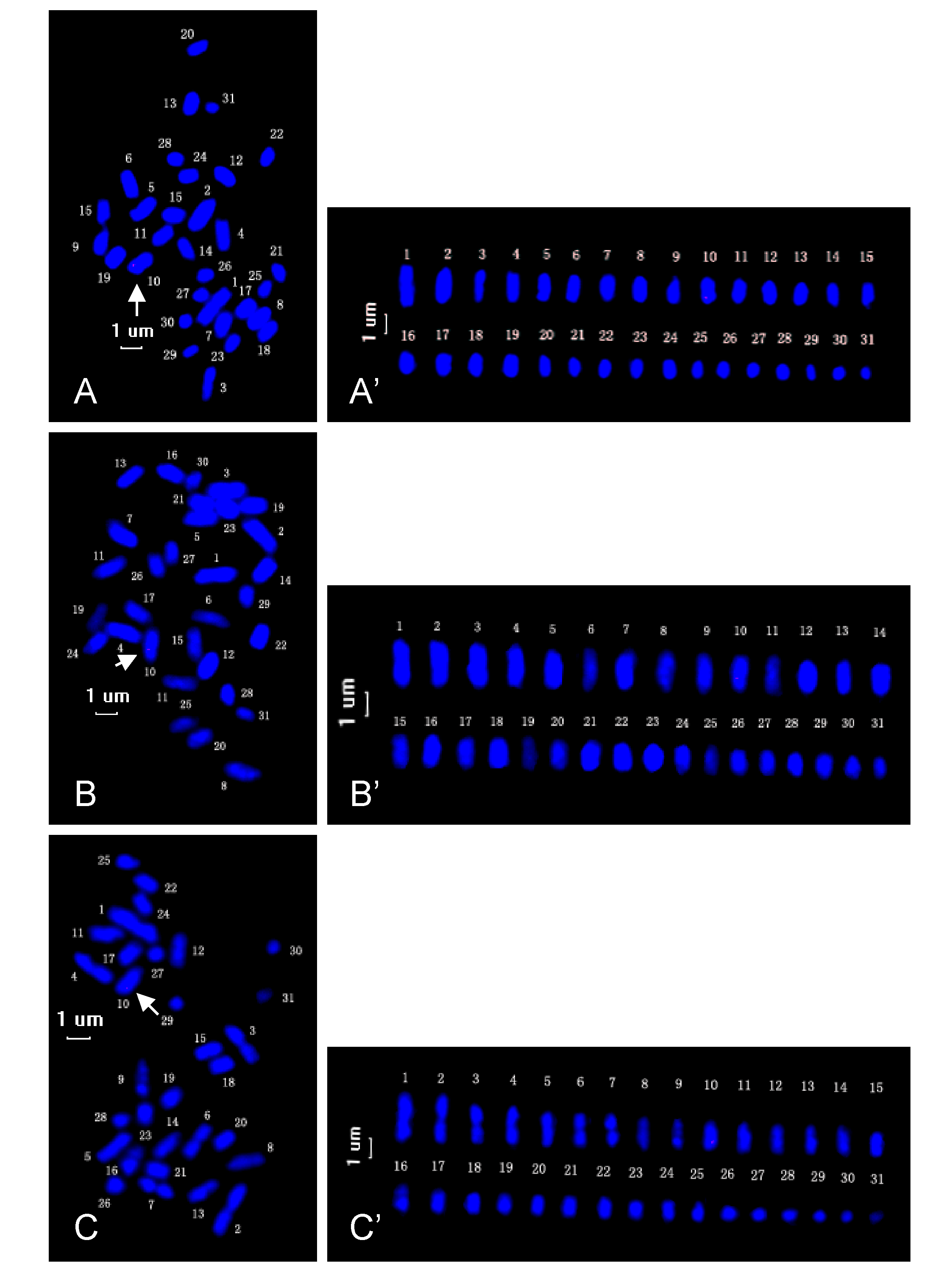

Supplement: Figure S1 — FISH images and their corresponding karyograms. FISH of the labeled FRML-494 marker (red) on metaphase chromosomes of the female gametophytes counterstained with DAPI (blue). A′, B′ and C′ are the ordered chromosomes of FISH images of A, B and C, respectively, prepared with Adobe Photoshop by decreasing size in length. Arrows indicating the localization of the FRML-494 marker on one chromosome of the female gametophytes. (TIF) [file pone.0048784.s001.tif]
